# Impact of Dyslipidemia on Allogeneic Transplantation Outcomes and Cardiovascular Mortality in Patients with Acute Leukemias in the Post-Transplant Cyclophosphamide Era

**DOI:** 10.3390/ph19040529

**Published:** 2026-03-25

**Authors:** Sema Seçilmiş, Burcu Aslan Candır, Uğur Hatipoğlu, Mert Seyhan, Bahar Uncu Ulu, Tuğçe Nur Yiğenoğlu, Dicle İskender, Merih Kızıl Çakar, Turgay Ulaş, Mehmet Sinan Dal, Fevzi Altuntaş

**Affiliations:** 1 Department of Hematology and Bone Marrow Transplantation Center, Etlik City Hospital, Ankara 06170, Türkiye; merihkizil@yahoo.com; 2 Department of Hematology and Bone Marrow Transplantation Center, University of Health Sciences, Ankara Oncology Training and Research Hospital, Ankara 06200, Türkiye; drburcuaslancandir@gmail.com (B.A.C.); ugurugur2192@gmail.com (U.H.); drseyhanmert@gmail.com (M.S.); baharuncu@gmail.com (B.U.U.); dr.nuryigenoglu@gmail.com (T.N.Y.); diclekoca@yahoo.com (D.İ.); turgayulas@yahoo.com (T.U.); dr.sinandal@gmail.com (M.S.D.); faltuntas@hotmail.com (F.A.); 3 Department of Internal Medicine, Division of Hematology, School of Medicine, Ankara Yıldırım Beyazıt University, Ankara 06760, Türkiye

**Keywords:** hematopoietic stem cell transplantation, dyslipdemia, post-transplant cyclophosphamide, acute leukemias, cardiovascular mortality

## Abstract

**Background/Objectives**: Allogeneic hematopoietic stem cell transplantation is associated with increased cardiovascular risk driven by endothelial dysfunction, chronic inflammation, and treatment-related metabolic disturbances, including dyslipidemia. In the contemporary era of post-transplant cyclophosphamide-based prophylaxis, the prognostic significance of dyslipidemia—particularly as assessed by non-HDL cholesterol—remains unclear. In this study, we aimed to compare the engraftment days, graft-versus-host disease (GVHD) development, relapse, overall survival rates, and cardiovascular mortality in patients using myeloablative/reduced intensity conditioning regimens with post-transplant cyclophosphamide (PTCy) 50 mg/kg/day for 2 days in patients with acute leukemias. **Methods**: A total of 95 adult patients with acute leukemias were included in their first remission who underwent matched sibling donor transplantation with PTCy (50 mg/kg on days +3 and +4). Patients were stratified according to pre-transplant non-HDL-C levels (<160 mg/dL vs. ≥160 mg/dL). Matched related donors were selected for the patients. All patients received either myeloablative or reduced-intensity conditioning based on EBMT criteria, with fludarabine-based combinations including busulfan, treosulfan, or TBI, along with ATLG administered at a total dose of 15 mg/kg. Peripheral blood stem cells were used for all transplants, and GVHD prophylaxis consisted of cyclosporine. **Results**: Platelet (median 13 vs. 14 days) and neutrophil (median 14 vs. 15 days) engraftment times and veno-occlusive disease (VOD) rates were comparable across groups (all *p* > 0.05); cumulative incidences of grade II–IV aGVHD at +100 days, grade III–IV aGVHD at +100 days, and moderate-severe cGVHD at 1 year, relapse-free survival, and non-relapse mortality at 1 year were comparable in two cohorts (all *p* > 0.05). GVHD-free/relapse-free survival (GRFS) at 1 year was also comparable across groups (*p* = 0.15). Median GRFS was 150 (95% CI: 120–330) days and 270 (95% CI: 154-not reached) days, respectively [HR was 0.68 (0.40–1.15), *p* = 0.15; GRFS at 1 year was 66.6% vs. 52.0%, respectively]. The groups were also comparable in terms of overall survival (OS). Follow-up ranged from 0.5 to 108 months, and median follow-up was 60 months in two cohorts. Median OS was not reached in non-HDL-C < 160 (95% CI: 70 months–not reached) and 67 months in non-HDL-C ≥ 160 groups (95% CI: 13 months–not reached) (Log rank = 0.21). No cardiovascular death events occurred during the follow-up period. **Conclusions**: In this homogeneous matched sibling donor transplant cohort with extended follow-up and uniform administration of post-transplant cyclophosphamide, cyclosporine-based GVHD prophylaxis, and anti-thymocyte lymphoglobulin (ATLG), pre-existing dyslipidemia was not associated with an adverse impact on GRFS, NRM, PFS, CMV reactivation, OS or long-term cardiovascular mortality.

## 1. Introduction

Allogeneic hematopoietic stem cell transplant recipients have an increased risk of cardiovascular disease related to chronic inflammation, endothelial dysfunction, immunosuppressive therapy, and frequent hypertriglyceridemia [1]. Endothelial dysfunction is a key mechanism underlying several complications after allogeneic hematopoietic stem cell transplantation (allo-HSCT), including graft-versus-host disease (GVHD) and transplant-associated microangiopathy. Conditioning regimens, immunosuppressive drugs, and systemic inflammation lead to endothelial activation and injury [2,3]. Dyslipidemia is also common after allo-HSCT, particularly in patients with GVHD. Although direct studies linking endothelial dysfunction and dyslipidemia in allo-HSCT are limited, both conditions share common inflammatory and immunometabolic pathways. Chronic inflammation and oxidative stress may simultaneously impair endothelial function and alter lipid metabolism [4].

Cardiovascular complications are increasingly recognized as an important cause of early and late non-relapse mortality after transplantation [5]. Beyond direct cyclophosphamide-related cardiotoxicity, traditional cardiovascular risk factors such as dyslipidemia and hypertension appear to contribute substantially to post-transplant cardiac events [6]. However, most of the existing evidence on dyslipidemia in allo-HSCT derives from heterogeneous cohorts treated with older GVHD prophylaxis strategies, limiting its relevance to contemporary transplant platforms. Emerging data from the PTCy era suggest that dyslipidemia may independently predict cardiovascular complications, thereby adversely influencing overall survival [7,8]. The widespread adoption of post-transplant cyclophosphamide (PTCy) has fundamentally altered transplant practice by improving GVHD control and modifying immune reconstitution, while also introducing potential cardiovascular effects related to cyclophosphamide exposure. In addition, PTCy-based approaches are increasingly applied in older and more comorbid patient populations, in whom baseline metabolic disturbances such as dyslipidemia are more prevalent. These observations highlight the need to better define the impact of lipid abnormalities on transplant outcomes and cardiovascular death in patients with acute leukemias undergoing allo-HSCT in the contemporary PTCy setting [9,10]. Taken together, these changes suggest that the prognostic significance of dyslipidemia may differ in the PTCy era compared with earlier transplant settings, representing an important and currently underexplored knowledge gap.

According to the European Society of Cardiology/European Atherosclerosis Society (ESC/EAS) and American College of Cardiology/American Heart Association (ACC/AHA) guidelines, non-HDL-C reflects the total burden of atherogenic lipoproteins, encompassing all apoB-containing particles, and is particularly useful in patients with secondary dyslipidemia [11,12]. In addition, non-HDL-C can be reliably measured in the non-fasting state and is less affected by postprandial triglyceride fluctuations compared with LDL-C, which enhances its practicality in routine clinical settings. Therefore, non-HDL-C constitutes a more appropriate marker for the assessment of dyslipidemia in this setting [1].

To date, no study has specifically investigated whether dyslipidemia affects transplantation outcomes in patients with acute leukemia receiving PTCy and cyclosporine alone as graft-versus-host disease (GVHD) prophylaxis. This is particularly important given the evolving transplant landscape and the potential interaction between PTCy-related effects and metabolic risk factors. Therefore, in this study, we aim to evaluate the impact of dyslipidemia on engraftment kinetics, GVHD incidence, relapse, overall survival (OS), and cardiovascular mortality in patients with acute leukemias.

## 2. Results

### 2.1. Patient Characteristics

The demographic and clinical features of the patients’ data are shown in Table 1. In non-HDL-C < 160; 25 (44.4%) patients had AML, 20 (55.6%) had ALL. Seven patients had RIC regimen and 38 patients had MAC. In the non-HDL-C ≥ 160 cohort; 25 (50%) patients had AML, 25 (50%) had ALL. Eight patients had RIC regimen and 42 patients had MAC.

### 2.2. Engraftment and VOD Information

In the non-HDL-C < 160 cohort and non-HDL-C ≥ 160 cohort, the median platelet engraftment times were 13 days and 14 days; median neutrophil engraftment times were 14 days and 15 days, respectively. Platelet and neutrophil engraftment were comparable across groups (*p* = 0.556 and *p* = 0.133, respectively). VOD rates were also similar across groups (*p* = 0.474) (Table 2).

### 2.3. Study Endpoints

**Figure 1:**
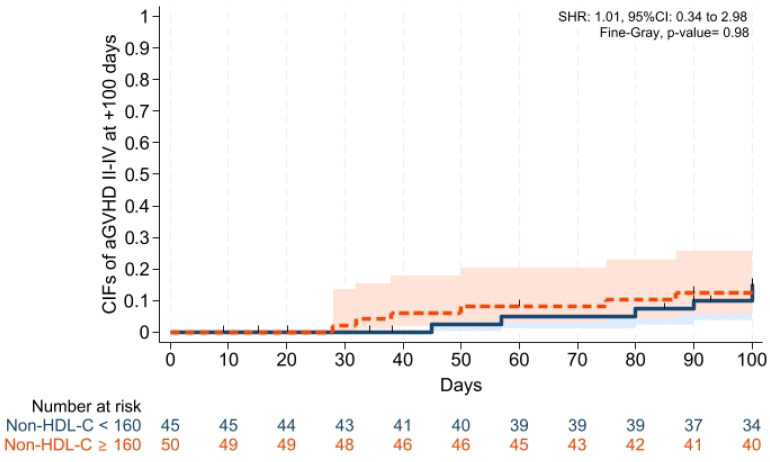
Cumulative incidence of grade II–IV aGVHD at day +100. Relapse and death were the competing risk factors. Solid and dashed lines represent the two study groups (non-HDL-C <160 mg/dL and ≥ 160 mg/dL, respectively). Shaded areas indicate 95% confidence intervals.

**Figure 2:**
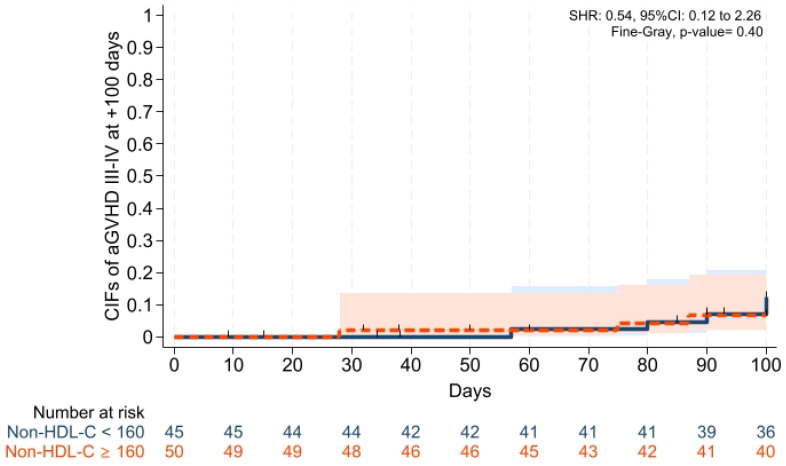
Cumulative incidence of grade III–IV aGVHD at day +100. Relapse and death were the competing risk factors. Solid and dashed lines represent the two study groups (non-HDL-C < 160 mg/dL and ≥ 160 mg/dL, respectively). Shaded areas indicate 95% confidence intervals.

**Figure 3:**
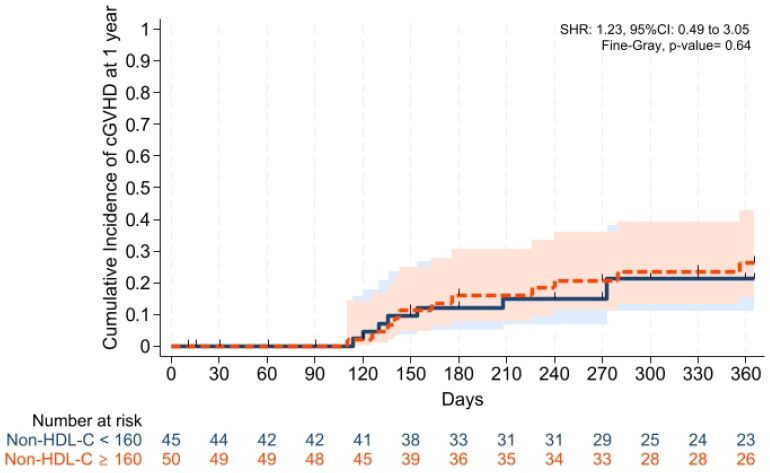
Cumulative incidence of moderate-to-severe cGVHD at 1 year. Relapse and death were the competing risk factors. Solid and dashed lines represent the two study groups (non-HDL-C < 160 mg/dL and ≥ 160 mg/dL, respectively). Shaded areas indicate 95% confidence intervals.

**Figure 4:**
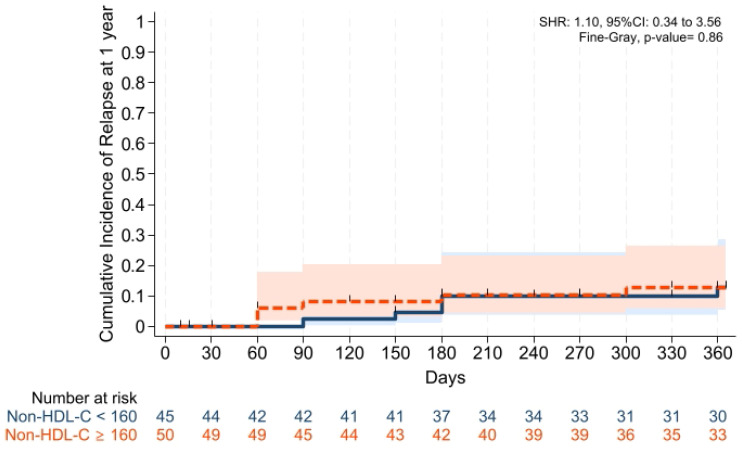
Cumulative incidence of relapse at 1 year. Death was the competing risk factor. Solid and dashed lines represent the two study groups (non-HDL-C < 160 mg/dL and ≥ 160 mg/dL, respectively). Shaded areas indicate 95% confidence intervals.

**Figure 5:**
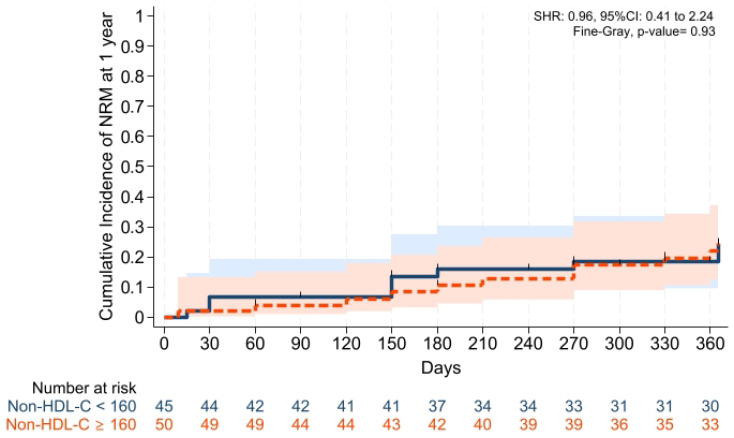
Cumulative incidence of NRM at 1 year. Relapse was the competing risk factor. Solid and dashed lines represent the two study groups (non-HDL-C < 160 mg/dL and ≥ 160 mg/dL, respectively). Shaded areas indicate 95% confidence intervals.

Cumulative incidences of grade II–IV aGVHD at +100 days, grade III–IV aGVHD at +100 days, and moderate-severe cGVHD at 1 year were comparable across groups (Figure 1, Figure 2 and Figure 3). Cumulative incidences of relapse and NRM at 1 year were also similar across groups (Figure 4 and Figure 5).

**Figure 6:**
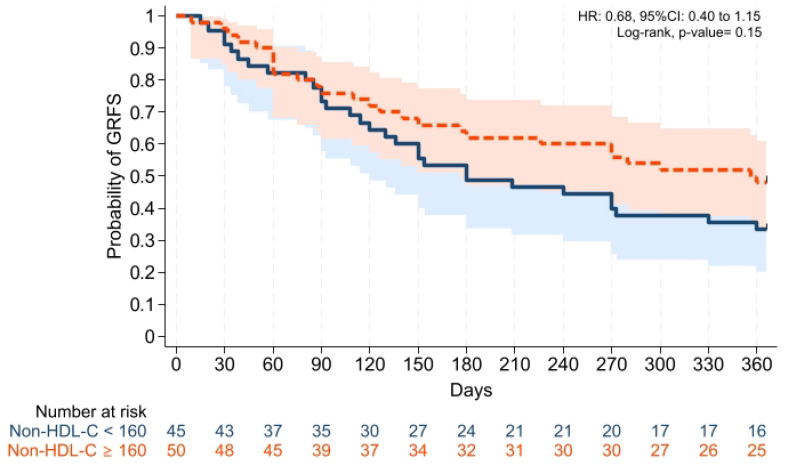
Kaplan–Meier plot for GRFS. Follow-up was limited to 1 year. Solid and dashed lines represent the two study groups (non-HDL-C < 160 mg/dL and ≥ 160 mg/dL, respectively). Shaded areas indicate 95% confidence intervals.

**Figure 7:**
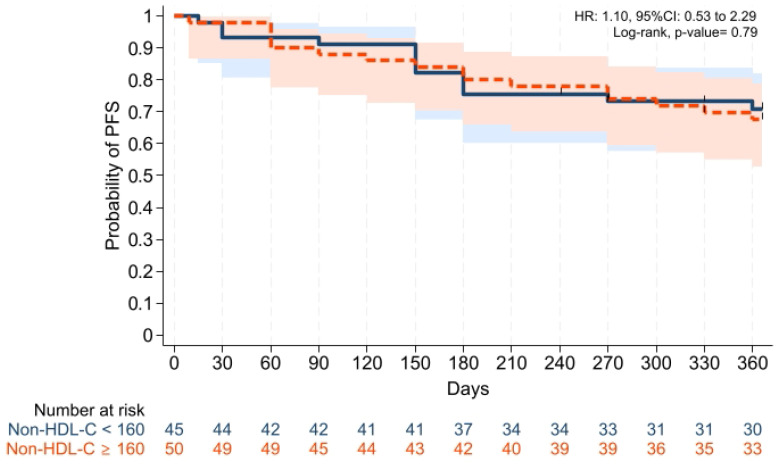
Kaplan–Meier plot for PFS. Follow-up was limited to 1 year. Solid and dashed lines represent the two study groups (non-HDL-C < 160 mg/dL and ≥ 160 mg/dL, respectively). Shaded areas indicate 95% confidence intervals.

**Figure 8:**
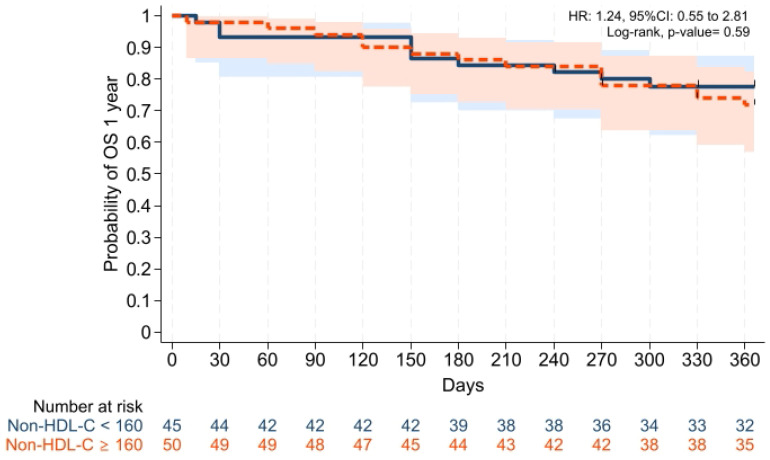
Kaplan–Meier plot for OS. Follow-up was limited to 1 year. Solid and dashed lines represent the two study groups (non-HDL-C < 160 mg/dL and ≥ 160 mg/dL, respectively). Shaded areas indicate 95% confidence intervals.

**Figure 9:**
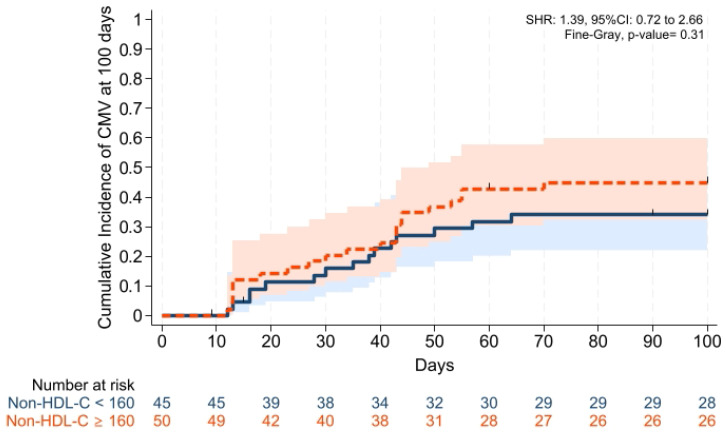
Cumulative incidence of post-transplantation CMV reactivation at 100 days. Solid and dashed lines represent the two study groups (non-HDL-C < 160 mg/dL and ≥ 160 mg/dL, respectively). Shaded areas indicate 95% confidence intervals.

**Figure 10:**
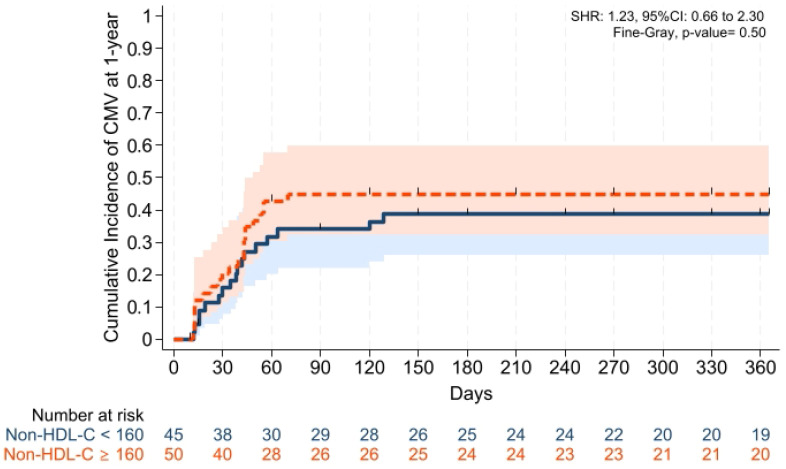
Cumulative incidence of post-transplantation CMV reactivation at 1 year. Solid and dashed lines represent the two study groups (non-HDL-C < 160 mg/dL and ≥ 160 mg/dL, respectively). Shaded areas indicate 95% confidence intervals.

GVHD-free/relapse-free survival (GRFS) at 1 year was also comparable across groups (*p* = 0.15). Median GRFS was 150 (95% CI: 120–330) days and 270 (95% CI: 154–not reached) days, respectively [HR was 0.68 (0.40–1.15), *p* = 0.15; GRFS at 1 year was 66.6% vs. 52.0%, respectively] (Table 3, Figure 6). Similarly, 1 year PFS and OS were comparable between the groups (*p* = 0.79 and *p* = 0.59, respectively) (Table 3, Figure 7 and Figure 8). The CI of post-transplantation CMV reactivation at 100 days and 1 year was comparable between the groups (both *p* > 0.05) (Figure 9 and Figure 10).

**Figure 11:**
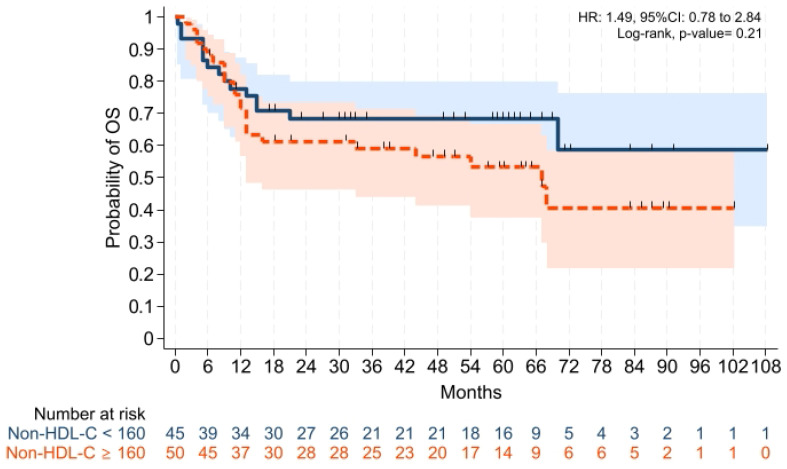
Kaplan–Meier plot for OS. Solid and dashed lines represent the two study groups (non-HDL-C < 160 mg/dL and ≥ 160 mg/dL, respectively). Shaded areas indicate 95% confidence intervals.

The groups were also comparable in terms of OS. Follow-up ranged from 0.5 months to 9 years, and median follow-up was 60 months in both cohorts (min–max 0.5–108 and 2.0–102 months, respectively). During the 9 year follow-up period, the overall mortality rate in the entire cohort was 41.1%. Mortality was 33.3% in the non-HDL-C < 160 group (15/45 patients), and 48.0% in the non-HDL-C ≥ 160 groups (24/50 patients). Median OS was not reached in non-HDL-C < 160 (95% CI: 70 months–not reached) and 67 months in non-HDL-C ≥ 160 groups (95% CI: 13 months–not reached) [HR was 1.49 (0.78–2.84), *p* = 0.21; OS at 9 year was 41.3% vs. 59.0%, respectively] (Table 3, Figure 11).

The causes of mortality did not differ significantly between the groups. Relapse was the leading cause of death in both cohorts, followed by sepsis and GVHD-related complications, and unknown causes (*p* = 0.330) (Table 2). Although cardiovascular mortality was predefined as a secondary outcome, cumulative incidence analysis could not be performed using competing-risk methodology because no cardiovascular death events occurred during follow-up.

## 3. Discussion

To our knowledge, this is the first study investigating whether dyslipidemia affects the transplantation outcomes, especially in leukemias receiving PTCy and cyclosporine alone for GVHD prophylaxis in patients with acute leukemias, and the main findings of our study were: (i) Platelet and neutrophil engraftment times and VOD rates were comparable across groups; (ii) CIFs of grade II–IV aGVHD at +100 days, grade III–IV aGVHD at +100 days, and moderate-severe cGVHD at 1 year were comparable between groups; (iii) CIFs of relapse and non-relapse mortality, and GRFS at 1 year were similar in two cohorts; (iv) 1 year PFS and OS were comparable between the groups; (v) post-transplantation CMV reactivation at +100 days and 1 year was comparable between the groups; and (vi) no significant difference was observed in terms of overall survival, and the cumulative incidence of cardiovascular mortality during follow-up was 0% in both groups.

Although lipid abnormalities are frequently observed in patients undergoing hematopoietic stem cell transplantation, data regarding the impact of pre-transplant dyslipidemia on transplantation outcomes are limited. Lin et al. evaluated lipid profiles both before and after transplantation and reported that lipid alterations, particularly decreased HDL-C levels, were associated with an increased risk of GVHD and inferior transplantation outcomes in allogeneic hematopoietic stem cell transplantation recipients [13]. Premstaller et al. reported that pre-transplant dyslipidemia was present in a substantial proportion of HSCT recipients and was independently associated with the development of post-transplant dyslipidemia. However, no significant association was observed between pre-transplant dyslipidemia and major transplantation outcomes, including GVHD, relapse, engraftment, or overall survival [14]. Similarly, in a retrospective study evaluating pre-transplant lipid parameters in allogeneic stem cell transplant recipients, no significant association was found between conventional pre-transplant dyslipidemia and key transplantation outcomes, including acute or chronic GVHD and overall survival [15]. Hatfield et al. also analyzed pretransplant systemic lipidomic profiles in allogeneic stem cell transplant recipients and demonstrated that specific lipidomic signatures were associated with post-transplant complications, particularly GVHD. The study did not specifically assess conventional pre-transplant dyslipidemia as a clinical risk factor for engraftment, relapse, or overall survival [16]. In our study, although pre-transplant lipid levels were measured, we evaluated the specific impact of pre-transplant dyslipidemia on transplantation outcomes in acute leukemia patients receiving PTCy and cyclosporine alone for GVHD prophylaxis, and observed that 100 day and 1 year transplant outcomes were comparable between the groups.

Cardiovascular complications have been increasingly recognized as an important late effect after allogeneic transplantation. Prior studies have demonstrated an elevated long-term risk of cardiovascular disease and mortality among hematopoietic cell transplant survivors, particularly in the presence of metabolic abnormalities and dyslipidemia [17,18,19,20]. A multicenter EBMT cohort found that arterial cardiovascular events occurred in long-term allogeneic transplant survivors, with a cumulative incidence of about 6% at 15 years post-transplant [17]. Moreover, long-term survivors have been shown to be at increased risk for cardiovascular diseases, likely driven by accelerated atherosclerosis and early development of cardiovascular risk factors after transplantation [19]. Meta-analytic data indicate that cardiovascular events remain a meaningful source of morbidity and mortality in this population, with long-term incidences observed beyond 100 days post-transplant [21]. Nevertheless, cardiovascular events typically emerge many years after transplantation and require large cohorts for adequate statistical power. In our study, no cardiovascular deaths were observed during follow-up, which precluded competing-risk analysis. This finding may be attributable to the relatively limited sample size, the younger transplant population, effective cardiovascular risk management during follow-up, or the predominance of other transplant-related causes of mortality. In addition, the outcomes of censored patients beyond the last follow-up were unknown, which may have led to an underestimation of late cardiovascular events. Therefore, our study is not powered to draw conclusions regarding the impact of non-HDL-C levels on cardiovascular mortality. Therefore, while no cardiovascular deaths were observed in this cohort during follow-up, this should be interpreted as an observation rather than evidence of absence of effect.

Certain limitations should be considered, including the retrospective, non-randomized study design and the single-center nature of the study. In addition, the final sample size of 95 patients was smaller than the 172 patients estimated to achieve 80% power for detecting the prespecified difference in 1 year GRFS between the non-HDL-C ≥ 160 mg/dL and <160 mg/dL groups. Therefore, the study is underpowered, and the absence of a statistically significant association between dyslipidemia and adverse outcomes may reflect a Type II error rather than definitive evidence of no effect. These findings should be interpreted with caution, and larger, adequately powered studies are needed to confirm the impact of dyslipidemia in this patient population.

In conclusion, in a highly homogeneous matched sibling donor transplant platform with a long-term follow-up of the patients, uniformly incorporating post-transplant cyclophosphamide, cyclosporine-based GVHD prophylaxis, and ATLG, pre-existing dyslipidemia was not associated with GRFS, OS, NRM, PFS, or CMV reactivation. No cardiovascular deaths were observed during the follow-up period; however, given the small sample size and median follow-up of 60 months, this should be interpreted as an observation rather than definitive evidence, and longer-term studies are warranted to assess potential cardiovascular effects. Competing-risk analyses further confirmed the absence of a clinically meaningful impact of dyslipidemia on transplant-related and disease-related outcomes. These findings suggest that, within a standardized and profoundly immunomodulated transplant setting, dyslipidemia does not constitute an independent determinant of long-term transplant outcomes. Our results support the notion that previously reported adverse associations may be driven by heterogeneity in transplant platforms and immunosuppressive strategies, and indicate that dyslipidemia alone may not be a major determinant of transplant decision-making or post-transplant risk stratification in similar settings.

## 4. Materials and Methods

### 4.1. Subjects

We conducted this retrospective non-randomized study and analyzed 95 patients who received their first transplantation for acute leukemias in their first remission and had PTCy dosing of PTCy 50 mg/kg/day for 2 days between 2014 and 2022. Patients were divided into two groups; patients who had non-HDL-C < 160 (*n* = 45), and non-HDL-C ≥ 160 (*n* = 50).

All patients included in the study were 18 or older. Exclusion criteria included patients with incomplete or missing key information, such as treatment details, engraftment data, GVHD status, relapse data, or survival outcomes. Patients were also excluded if they did not receive the specified conditioning regimens (myeloablative or reduced intensity). Additional exclusions applied to patients with other hematologic or non-hematologic malignancies outside the study criteria, those who had undergone prior allogeneic hematopoietic stem cell transplantation, and those treated with experimental therapies, investigational drugs, receiving cholesterol-lowering medications at the time of transplantation or adjunctive treatments not included in the study protocol (e.g., post-transplant maintenance therapies). Furthermore, patients who received stem cells from sources not specified in the study (e.g., umbilical cord blood or ex vivo T-cell-depleted grafts) were excluded.

Our center, Ankara Oncology Hospital, has standard operation procedures using ATLG (Grafalon®, Neovii Biotech, Rapperswil, Switzerland) for all conditioning regimens, and all patients receive PTCy. All the patients had cardiology, pulmonology, gynecology (for females), psychiatry, and dental evaluation for transplant preparation.

### 4.2. Ethics Approval and Consent

Ethical approval was obtained from Ankara Oncology Training and Research Hospital in accordance with the Declaration of Helsinki (approval number: 2022-06/1920), and written informed consent was obtained from all patients authorizing the use of their data for research purposes prior to the transplantation.

### 4.3. Donor Selection

Matched related donors were selected for the patients.

### 4.4. Conditioning Regimens and Transplantation Procedure

All of the laboratory values obtained prior to the initiation of the conditioning regimen and peripheral collected stem cells were used for all transplants. All patients received MAC or RIC conditioning regimens according to the EBMT definitions [22,23]: fludarabine 150 mg/m^2^, busulfan 12.8 mg/kg, treosulfan 42 gr/m^2^, and fractionized 12 Gray total body irradiation (TBI) for MAC, and fludarabine 150 mg/m^2^, busulfan 6.4 or 9.6 mg/kg, treosulfan 30 or 36 gr/m^2^, and fractionized 8 Gray TBI were administered for RIC regimens. Patients with AML received fludarabine/busulfan/ATLG or fludarabine/treosulfan/ATLG, whereas patients with ALL received fludarabine/TBI/ATLG combinations.

ATLG (Grafalon) was administered −5 to −3 at a total dose of 15 mg/kg (5 mg/kg for 3 days) for all patients based on our center’s standard operation procedures. All transplants were performed using doses of PTCy 50 mg/kg twice daily on days +3 and +4. G-CSF was given to the patients when needed based on clinician discretion.

### 4.5. Definitions

Risk stratification for non-HDL cholesterol is derived from the NCEP ATP III classification and the ESC/EAS guideline approach defining non-HDL-C targets as 30 mg/dL above LDL-C goals. Accordingly, non-HDL-C levels <130 mg/dL indicate low risk, 130–159 mg/dL intermediate risk, ≥160 mg/dL high atherogenic risk, and ≥190 mg/dL very high risk [11,24]. Given accumulating evidence, we selected non-HDL-C as the primary parameter for our analysis. Although all measurements were performed in the fasting state prior to transplantation, leukemia-related metabolic disturbances and triglyceride abnormalities may compromise the reliability of LDL-C. Therefore, non-HDL-C was considered a more robust and clinically meaningful marker of atherogenic burden in this population.

Neutrophil engraftment was defined as a measurable neutrophil count > 0.5 × 10^9^/L for three consecutive days. Non-relapse mortality (NRM) was defined as deaths not related to relapse. Graft-versus-host relapse-free survival (GRFS) was described as the time elapsed between transplantation and aGVHD, cGVHD, relapse, death, or last contact. Progression-free survival (PFS) was estimated as the time between transplantation, relapse, death, or last contact. OS was defined as the time between the transplantation and death or last contact.

### 4.6. GVHD Prophylaxis and Assessment

Based on standard operation procedure in our center, cyclosporine alone was administered for GVHD prophylaxis for patients who had a matched sibling donor. The physician confirmed the diagnosis of aGVHD based on clinical, radiologic, and laboratory results. The Mount Sinai Acute GVHD International Consortium (MAGIC) standards were used to assess the staging and grading of aGVHD [25,26]. National Institutes of Health (NIH) consensus criteria were used for the diagnosis and staging/grading of cGVHD [27].

### 4.7. Endpoints

The primary endpoint of this analysis was GRFS at 1 year. Secondary endpoints included OS, cumulative incidence functions (CIF) of grade II–IV aGVHD at day +100, grade III–IV aGVHD at day +100, moderate-severe cGVHD at 1 year, relapse at 1 year, NRM at 1 year and cardiovascular mortality during follow-up.

### 4.8. Sample Size Calculation

To our knowledge, no prior study has specifically evaluated the impact of dyslipidemia on 1 year GRFS in patients undergoing matched sibling donor HSCT with PTCy-based prophylaxis. Therefore, previously published GRFS outcomes in comparable transplant platforms were used as reference values. Previous studies have reported 1 year graft-versus-host disease-free, relapse-free survival (GRFS) rates in allogeneic HCT with PTCy-based GVHD prophylaxis ranging from ~43% to ~55% in randomized trials and matched sibling donor settings, supporting its use as a reference for benchmarking outcomes [28,29]. Therefore, the sample size was calculated to compare 1 year GRFS between the non-HDL-C ≥ 160 mg/dL group (expected GRFS 50%) and the non-HDL-C < 160 mg/dL group (expected GRFS 30%), assuming a two-sided α of 0.05, 80% power, and a 1:1 allocation ratio. Under an exponential survival assumption, this corresponds to a hazard ratio (HR) of 0.58. Using the Schoenfeld method for the log-rank test, 103 events were required. Given an average expected event rate of 60%, the total required sample size was estimated to be 172 patients (86 per group).

### 4.9. Statistical Analysis

IBM SPSS Statistics version 26 and Stata SE 19.5 for Windows (StataCorp, College Station, TX, USA) were used for statistical analyses. Continuous variables were expressed as median and interquartile ranges (25% and 75%), whereas categorical variables were expressed as numbers and percentages. The Kolmogorov–Smirnov test was applied to test for data distribution. An independent sample *t*-test, Mann–Whitney U test, and Pearson’s chi-square/Fisher’s exact tests were used for numerical and categorical data for comparisons. Reverse Kaplan–Meier analysis was used for median follow-up times. Competitive risk analyses were performed for all CIF calculations. In the CIF analyses of grade II–IV aGVHD at day +100, grade III–IV aGVHD at day +100, and moderate-severe cGVHD at day +100, relapse and death were regarded as the competitive risk factors. On the other hand, death was the only competitive risk factor for CIFs of relapse at 1 year, and NRM at 1 year. Patients who did not experience failure events within 100 days and 1 year for the cumulative analyses of grade II–IV aGVHD, grade III–IV aGVHD, and moderate-severe cGVHD, and CIFs of relapse and NRM, GRFS, PFS, and OS, respectively, were censored. The cumulative incidence of cytomegalovirus (CMV) reactivation by day 100 and 1 year were estimated using competing-risk methods, with death and relapse treated as competing events. For CIFs of CMV reactivation, only the first event was considered. Patients who experienced relapse or death before CMV reactivation were treated as having a competing event and were not counted as CMV events, even if CMV reactivation occurred later. The CIFs of cardiovascular mortality was also planned to be estimated using competing-risk methodology. Fine-Gray’s test was performed to determine the differences in the CIFs between groups. Confidence intervals (CIs) were used as an estimate of variation within each group. Sub-distributional, cause-specific hazard ratios (HRs) with corresponding CIs were presented. Survival function was assessed by the Kaplan–Meier method. Groups were compared by the log-rank test. Variance was assessed indirectly via the proportional hazards assumption. HRs with CIs were estimated by Cox proportional regression analyses. A two-sided *p*-value of ≤0.05 was considered statistically significant.

## Figures and Tables

**Table 1:** Demographic and Clinical Features of the Patients.

Variables	Non-HDL-C < 160 (*n* = 45)	Non-HDL-C ≥ 160 (*n* = 50)	*p*
Age, years	40 (27–49)	41 (32–52)	0.315 ^a^
Gender (F/M)	25/20	27/23	0.879 ^b^
BMI	25.91 (23.35–29.35)	26.55 (23.75–30.84)	0.301 ^a^
Leukemia subtypes AML ALL	25 (44.4) 20 (55.6)	25 (50.0) 25 (50.0)	0.588 ^b^
ECOG, 0/1	30/15	34/16	0.890 ^b^
Transplant Intensity, RIC/MAC	7/38	8/42	0.953 ^b^
CD34+ cell account, 10^6^/kg	7.50 (6.62–7.91)	7.32 (6.63–7.96)	0.631 ^c^
Blood group incompatibility None Major Minor Bidirectional	25 (55.6) 10 (22.2) 9 (20.0) 1 (2.2)	25 (50.0) 14 (28.0) 8 (16.0) 3 (6.0)	0.690 ^b^
Patient TC, mg/dL	163 (146–179)	240 (224–270)	<0.001 ^a^
Patient TG, mg/dL	195 (111–264)	243 (164–347)	0.009 ^c^
Patient LDL-C, mg/dL	98 (71–106)	145 (130–169)	<0.001 ^c^
Patient HDL-C, mg/dL	38 (28–47)	44 (36–55)	0.022 ^a^
Patient Non-HDL-C, mg/dL	125 (110–141)	196 (180–225)	<0.001 ^c^
Donor age, years	34 (24–44)	39 (33–50)	0.018 ^a^
Donor gender (F/M)	14/31	22/28	0.196 ^c^
Donor TC, mg/dL	173 (147–202)	182 (158–221)	0.174 ^c^
Donor TG, mg/dL	97 (66–146)	121 (79–175)	0.086 ^c^
Donor LDL-C, mg/dL	105 (88–123)	117 (89–146)	0.040 ^a^
Donor HDL-C, mg/dL	45 (40–50)	44 (38–52)	0.302 ^c^
Donor Non-HDL-C, mg/dL	131 (106–154)	136 (114–173)	0.112 ^c^

Independent sample *t*-test ^a^, Pearson chi-square ^b^, Mann–Whitney U ^c^; continuous variables expressed as median and interquartile range; categorical variables were expressed as numbers and percentages. Abbreviations: F/M: female/male; BMI: body mass index; AML: acute myeloid leukemia; ALL: acute lymphoblastic leukemia; ECOG: Eastern Cooperative Oncology Group; RIC: reduced-intensity conditioning; MAC: myeloablative conditioning; TC: total cholesterol; TG: Triglyceride; LDL-C: low-density lipoprotein cholesterol; HDL-C: high-density lipoprotein cholesterol.

**Table 2:** Transplant Outcomes.

Variables	Non-HDL-C < 160 (*n* = 45)	Non-HDL-C ≥ 160 (*n* = 50)	*p*
Neutrophil engraftment, days	14 (13–15)	15 (14–16)	0.133 ^a^
Platelet engraftment, days	13 (12–17)	14 (12–17)	0.556 ^a^
Post-transplant VOD	1/44	0 (50)	0.474 ^b^
Cause of mortality	15	24	
Sepsis	7	7	
Relapse	7	12	0.330 ^c^
GVHD Unknown	1 -	2 3	

Mann–Whitney U ^a^, Fisher’s exact test ^b^, Pearson chi-square ^c^; continuous variables expressed as median and interquartile range; categorical variables were expressed as numbers. Abbreviations: VOD: veno-occlusive disease; GVHD: graft-versus-host disease.

**Table 3:** Comparisons of clinical outcomes.

Outcome	Non-HDL-C < 160 % (95% CI)	Non-HDL-C ≥ 160 % (95% CI)	*p*
Number	29	41	
Cumulative incidence of grade II–IV aGVHD at day +100, %	9.8 (3.8–24.1)	12.4 (5.8–25.6)	0.98
Cumulative incidence of grade III–IV aGVHD at day +100, %	7.2 (2.4–20.1)	6.6 (2.2–19.1)	0.40
Cumulative incidence of moderate-severe cGVHD at 1-year, %	21.0 (11.0–38.0)	26.0 (15.0–42.0)	0.65
Cumulative incidence of relapse at 1-year, %	12.9 (5.5–28.0)	12.8 (5.9–26.0)	0.86
Cumulative incidence of NRM at 1-year, %	18.3 (9.6–33.5)	22.0 (12.4–37.2)	0.93
Cumulative incidence of CMV reactivation at day +100, %	34.0 (22.1–49.9)	44.9 (32.3–59.8)	0.31
Cumulative incidence of CMV reactivation at 1-year, %	38.7 (26.1–54.7)	44.9 (32.3–59.8)	0.50
GRFS at 1 year, %	66.6 (52.9–79.8)	52.0 (39.0–66.3)	0.15
PFS at 1 year, %	70.8 (55.1–81.9)	67.7 (52.8–78.8)	0.79
OS at 1 year, %	77.5 (62.3–87.2)	71.7 (57.1–82.2)	0.59
OS at 9 years, %	41.3 (23.6–65.1)	59.0 (41.5–78.1)	0.21

Abbreviations: GVHD: graft-versus-host disease; NRM: non-relapse mortality; CMV: cytomegalovirus; GRFS: GVHD relapse-free survival; PFS: progression-free survival; OS: overall survival.

## Data Availability

The data presented in this study are available on request from the corresponding author. The data are not publicly available due to ethical and privacy restrictions.
